# Fermented Dairy Consumption and Metabolic Syndrome in Finnish Men and Women

**DOI:** 10.1016/j.tjnut.2026.101464

**Published:** 2026-03-02

**Authors:** Miika Wynne-Ellis, Tuula Tuure, Satu Männistö, Niina E Kaartinen, Mirkka Maukonen

**Affiliations:** 1 Department of Public Health and Welfare, Finnish Institute for Health and Welfare, Helsinki, Finland; 2 Department of Food and Nutrition, University of Helsinki, Helsinki, Finland; 3 Valio Ltd., Helsinki, Finland; 4 Folkhälsan Research Center, Helsinki, Finland

**Keywords:** fermented dairy, metabolic syndrome, dietary patterns, men and women, cardiometabolic health

## Abstract

**Background:**

Metabolic syndrome (MetS) is a cluster of cardiovascular disease risk factors, with diet playing a key role in its prevention. Fermented dairy products may influence MetS risk, with possible sex-specific associations.

**Objectives:**

We examined whether fermented dairy consumption was associated with MetS and its components in Finland. Associations with nonfermented and total dairy were also evaluated.

**Methods:**

This cross-sectional study included 5096 adults (44% men) from the National FinHealth 2017 study. Diet was assessed using a validated food frequency questionnaire. MetS was defined by Joint Interim Statement 2009 criteria. Logistic regression and analysis of covariance were adjusted for confounders.

**Results:**

MetS prevalence was 42.5% in men and 34.1% in women. In men, the highest quintile of fermented dairy (excluding cheese) consumption was associated with lower odds of MetS [odds ratio (OR): 0.72, 95% confidence interval (CI): 0.53, 0.98; *P*-trend = 0.018]. In women, compared with the lowest quintile, the highest quintile of cheese consumption showed inverse association and evidence of nonlinearity with MetS (OR: 0.73, 95% CI: 0.54, 1.00; *P*-trend = 0.046, quadratic term *P* value < 0.001). The nonfermented and total dairy consumption did not show significant associations with MetS in men; however, in women, the highest consumption was associated with higher odds of MetS. Among MetS components, fermented dairy was associated with lower triglycerides (TGs), and cheese with higher high-density lipoprotein (HDL) cholesterol and lower TGs in women. Nonfermented dairy was associated with adverse HDL profiles in both sexes. Dose-response analyses were consistent with the quintile-based findings, and quadratic terms indicated potential nonlinear associations.

**Conclusions:**

In this cross-sectional analysis, associations between dairy consumption and MetS varied by sex and dairy subtype. An inverse association was observed for fermented dairy in men and for cheese in women; however, several associations were null, and in women, higher odds of MetS were observed mainly at the highest consumption of total and nonfermented dairy.

## Introduction

Metabolic syndrome (MetS) comprises a cluster of cardiovascular disease (CVD) risk factors, including dyslipidemia, hypertension, obesity, and impaired glucose tolerance [1]. The prevalence of MetS varies depending on the definition used, ranging from 24% to 65% in women and 43% to 78% in men across Europe and Nordic countries [2,3]. MetS is a significant precursor to CVD and type 2 diabetes mellitus [4,5]. Lifestyle interventions, such as weight reduction, increased physical activity, and improved dietary quality, can prevent and reverse MetS [5]. Given the rising prevalence of MetS, identifying dietary factors, including the potential role of dairy, is crucial for mitigating its onset and associated risks.

Recent systematic reviews and meta-analyses of cross-sectional and longitudinal studies have suggested that dairy products, particularly fermented varieties such as yogurt, kefir, and fermented milk, may have neutral or protective associations with MetS [[6], [7], [8], [9], [10]]. The most consistent associations reported in reviews and meta-analyses are with blood lipids and hypertension. Evidence from controlled trials further strengthens these observations. In a 21-wk crossover study involving 29 healthy females, daily 300 g consumption of yogurt increased HDL cholesterol and improved the LDL/HDL ratio [11]. In a randomized placebo-controlled study, daily consumption of fermented milk for 21 wk lowered blood pressure in hypertensive individuals [12]. However, evidence on cheese and its relationship with MetS components remains inconclusive, indicating a need for further investigation [10,[13], [14], [15]].

Although previous studies have explored the association between fermented dairy consumption and metabolic health, only 2 studies have examined sex-specific differences in these associations, revealing intriguing sex-specific responses. A French cohort study, including 288 men and 300 women, found that in men, consumption of milk, yogurt, and cottage cheese was associated with lower blood glucose and higher HDL cholesterol levels over a 5-y follow-up [16]. In contrast, among women, baseline dairy consumption was positively associated with BMI and waist circumference, and inversely with HDL, but no significant longitudinal associations with MetS components were observed [16]. In a Korean cohort study of 2859 men and 2651 women, the inverse association between yogurt and low HDL cholesterol was found only in women [17]. However, the overall risk for MetS was lower in both sexes [17]. These findings underscore the potential for sex-specific responses to different dairy products and highlight the importance of further research with sex-stratified analyses.

Also, dairy consumption varies across cultures, both in quantity and type. For example, in Korean adults, the highest consumption category was just 1 serving per day, which is the same as the lowest consumption in the European population [16,17]. Studying these associations in a proper cultural context is important for understanding associations in populations with similar dietary habits. Currently, there is no research on the associations between dairy consumption and MetS in Nordic countries.

Our study aimed to address this gap in the literature, offering nuanced information on the associations of fermented dairy and MetS within a Nordic population characterized by high dairy consumption and notable variation in individual dairy consumption. This population-based study investigated the associations between fermented dairy products [yogurt, sour milk, quark, and viili (Finnish fermented milk product)], cheese (aged cheese, processed cheese, and spreadable cheese), and MetS and its components in the Finnish general population. By addressing sex-specific differences, nonlinearity, focusing on cheese separately, and comparing fermented to nonfermented products, this research provides novel insights and addresses a critical gap in the literature, offering valuable implications for dietary recommendations at both the individual and population levels.

## Methods

### Study population

This study is a cross-sectional analysis of the National FinHealth 2017 data [18]. The National FinHealth 2017 Study examines the health, well-being, and functional capacity of the Finnish adult population. A nationally representative sample of 10,247 adults residing in mainland Finland was drawn from the population register by 2-stage cluster sampling. Individuals within the sample received an invitation to a health examination, including anthropometric measurements, blood pressure, blood samples, and self-administered questionnaires. Of the invited, 58% participated in the health examination.

The food frequency questionnaire (FFQ) was administered to participants attending the health examination. In total, 5302 FFQs were returned (89% return rate). Participants with multiple blank rows on the FFQ were excluded (*n =* 110). When an FFQ was returned in both paper and electronic format (*n =* 9), only the first returned FFQ was retained (paper or electronic). Participants with implausible energy intake were excluded by trimming the lowest and highest 0.5% of daily energy intake separately in men and women (*n =* 51), following standard methods [19]. Participants who withdrew consent (*n =* 7) were also excluded. In addition, pregnant participants and those with missing MetS component data (*n =* 29) were excluded. After these exclusions, 5096 participants (2846 women and 2250 men) remained for the analyses (Supplementary Figure 1).

The study protocol was conducted in accordance with the Declaration of Helsinki. The Coordinating Ethics Committee at the Hospital District of Helsinki and Uusimaa approved all procedures involving human participants (Reference 37/13/03/00/2016). All participants provided written informed consent.

### MetS assessment

MetS was defined using the Joint Interim Statement (JIS) 2009 criteria [20], which require ≥3 specific components to be met, and was selected based on its strong correlation with other diagnostic methods in the Finnish population, as demonstrated by Haverinen et al. [3]:

1) Increased waist circumference (European population-based; men: ≥94 cm, women: ≥80 cm).
2) Triglycerides (TGs) ≥1.7 mmol/L (150 mg/dL).
3) HDL; men: <1.03 mmol/L (40 mg/dL), women <1.3 mmol/L (50 mg/dL).
4) Fasting glucose ≥5.6 mmol/L (100 mg/dL) or medication.
5) Blood pressure ≥130/85 mm Hg or medication.

Blood samples were collected from participants who had fasted and refrained from heavy exercise for ≥4 h before the examination. Samples were frozen on site and transported to the accredited biochemistry laboratory of the Finnish Institute for Health and Welfare, where HDL cholesterol and TG were analyzed using the enzymatic Abbott method (Abell–Kendall verification). Glucose was measured by the enzymatic hexokinase Abbott (NIST SRM 956).

During health examinations, trained research staff measured participants’ height (cm), weight (kg), waist circumference (cm), and blood pressure (mm Hg) following standard protocols [21]. BMI was calculated as weight divided by height squared (kg/m^2^). Blood pressure was measured 3 times using a mercury sphygmomanometer on the right arm while participants were seated, and the mean of the second and third readings was recorded. For the analysis, systolic and diastolic blood pressure (DBP) were calculated into mean arterial pressure (MAP), which considers both systolic and DBP, by the following formula:

MAP=[SBP+(2×DBP)]/3

where MAP = mean arterial pressure, SBP = systolic blood pressure, and DBP = diastolic blood pressure [22].

Information regarding the current use of antihypertensive medication and diabetes medication (categorized by type and frequency of use) was collected through self-administered questionnaires. The current use of antihypertensive medication was defined as medication taken within the past 7 d, whereas the current use of diabetes medication included the use of either insulin or tablets.

### Dietary assessment

Data on habitual diet for the preceding 12 mo were collected using a validated semiquantitative 134-item FFQ, a tool that has undergone repeated validation and update in the Finnish adult population [23,24]. This FFQ inquired about the consumption of commonly consumed foods, mixed dishes, and beverages in Finland [25]. Participants reported their food consumption frequency using 10 categories ranging from none to ≥6 per day, with each item assigned a fixed portion size specific to sex (e.g., glass, slice, and volume). Mean daily food consumption and nutrient intake in grams per day, as well as energy intake (in kilojoules per day), were calculated using in-house software Finessi and the Finnish Food Composition Database Fineli [26]. In our analysis, the main food groups used were fermented dairy (yogurt, sour milk, viili, and quark) and cheese (aged cheese, processed cheese, and spreadable cheese). For comparison, we also examined nonfermented dairy (milk) and total dairy, which included all previously mentioned dairy groups. Mean consumptions for each dairy product are presented in Supplementary Table 1.

### Potential confounding factors

Derived from the sampling frame, participant demographics, including age and sex, were obtained. Self-administered questionnaires covered education, smoking habits, and leisure-time physical activity. Education status was classified into 3 levels: low, middle, and high, based on the number of years of education. For education status, participants were categorized into sex- and birth cohort–specific educational tertiles based on self-reported total years of education, to account for secular trends in the expansion of the education system and the increases in mean education duration over time. The self-administered questionnaire collected information on participants’ smoking history and current habits. For the analysis, all participants were categorized into 2 groups: current smokers and nonsmokers who had not smoked in the last 6 mo. Leisure-time physical activity over the past 12 mo was assessed using a 4-level scale: no physical activity (reading, watching television), light physical activity for ≥4 h per week (walking, gardening), moderate to vigorous physical activity for ≥3 h per week (running, swimming), and competitive athlete (heavy sports several times a week). The last 2 categories were combined due to low participation in the latter.

### Statistical analyses

All statistical analyses were performed using IBM SPSS Statistics for Windows, Version 25.0 (IBM Corp., Armonk). The significance level was set at *P* < 0.05 for all tests. Interaction by sex (male/female) and dairy groups on MetS risk was tested, and statistically significant interactions with fermented, total dairy, cheese, and nonfermented dairy consumption for MetS were observed (all *P* values <0.05). Therefore, all analyses, including MetS components, were stratified by sex.

Binary logistic regression was employed to examine the association between dairy consumption and the prevalence of MetS (yes/no). Dairy consumption was categorized into quintiles for *1*) fermented dairy excluding cheese, *2*) cheese, *3*) nonfermented dairy, and *4*) total dairy. For trend tests, each participant was assigned the quintile-specific median consumption, which was entered as a continuous predictor in the logistic model; the Wald *P* value for this coefficient was taken as the *P*-trend.

Additionally, we investigated nonlinear associations, demonstrated in previous cross-sectional studies [6]. Nonlinearity was evaluated by additionally including the mean-centered median and its squared term in the same model; the Wald *P* value for the squared term provided the *P*-quadratic (departure from linearity). For nonlinear analyses in continuous MetS components, we used linear regression with the same approach (median and median^2^ entered simultaneously), interpreting the *P* values in the same manner.

Logistic regression was also performed using 200 g/d for fermented, nonfermented, and total dairy, and 30 g/d for cheese, to assess dose-response associations. These amounts, used in a previous study with similar methods [27], approximate 1 SD of consumption in our data. Dose-response estimates are expressed as the change in outcome per 200 g/d (or 30 g/d for cheese), shown as odds ratio (OR) ± 95% confidence interval (CI) for MetS and as mean change ± 95% CI for individual MetS components. Model discrimination was evaluated using receiver operating characteristic analysis and the AUC, and model fit was assessed using the Hosmer–Lemeshow test and Nagelkerke’s *R*^2^.

To evaluate the association between dairy consumption and individual MetS components (systolic/DBP, fasting glucose, TGs, HDL cholesterol, and waist circumference), analysis of covariance was used. Dairy consumption (in quintile) was entered as a categorical factor, whereas the MetS components were continuous outcomes. We computed estimated marginal means (adjusted means) for each quintile. Trend *P* values across quintiles were obtained by modeling the quintile medians as a continuous variable. Linear regression was also performed to assess potential dose-response associations.

We used 2 main models to adjust for potential confounders. We selected covariates based on the literature, identifying both established and potential risk factors for MetS [1,4,5].

Model 1: adjusted for age (y) and total energy intake (kJ/d).

Model 2: model 1 further adjusted for physical activity (inactive, moderately active, highly active), smoking status (current smoker compared with nonsmoker in the past 6 mo), alcohol intake (ethanol g/d), sucrose intake (g/d), SFA intake (g/d), and fruit/berry/vegetable consumption (g/d).

As dairy is the primary source of SFAs in Finland, we conducted subanalyzes of high-fat and low-fat cheese among men and women to examine whether the associations with MetS differed by fat content.

An intermediate model, which included all covariates from model 2 except sucrose, fruits/berries/vegetables, and SFAs, was also initially tested. The results did not differ meaningfully from those of model 2, with a difference between means of <5%. Therefore, it was omitted from the main results.

We conducted an additional sensitivity analysis by excluding participants who were taking medications that could directly influence MetS, specifically hypertension medication and diabetes medication. Although this exclusion slightly attenuated some estimates, the overall associations remained robust, and the interpretation of the results was unchanged (data not shown).

We assessed lipid-lowering therapy with a dichotomous variable derived from the questionnaire item “Has a physician prescribed lipid-lowering medication?” JIS-2009 notes that fibrates and niacin affect TGs and HDL; however, these agents are rarely used in Finland. Because we could not verify whether any participants were on these HDL/TG-targeting therapies, we conducted sensitivity analyses excluding all participants who reported lipid-lowering medication and, separately, repeated all models with adjustment for lipid-lowering medication. In both cases, the results did not change meaningfully.

## Results

### Baseline characteristics for the study population

Men and women had significant differences in all MetS components, dietary, and confounding factors, except age and education (Table 1). Approximately 2 of 5 men and one-third of women had MetS. Compared with women, men had higher consumption of every dietary factor except fruits, berries, and vegetables (389 g/d for men compared with 481 g/d for women), as well as fermented dairy products (162 g/d for men compared with 194 g/d for women).

**TABLE 1:** Population characteristics for men and women.

Characteristic	Men (*n =* 2250)	Women (*n =* 2846)	*P* value∗∗∗∗
Confounding factors			
Age (y)	56 (16)	56 (16)	0.72
Highly educated participants^1^ (%)	37	34	0.06
Physically inactive participants (%)	22	26	<0.001
Currently smoking participants^2^ (%)	19	15	<0.001
Energy (kJ/d)	9736 (3280)	7933 (2648)	<0.001
Alcohol (ethanol g/d)	10.6 (16.2)	3.9 (6.5)	<0.001
Sucrose (g/d)	40.6 (24.3)	35.8 (19.6)	<0.001
SFAs (g/d)	36.1 (14.9)	28.8 (11.5)	<0.001
Fruits, berries, and vegetables (g/d)	389.4 (236.5)	481.1 (281.6)	<0.001
Dairy consumption			
Fermented dairy^3^ (g/d)	162.2 (193.2)	194.7 (203.7)	<0.001
Cheese^4^ (g/d)	52.4 (42.2)	46.3 (38.6)	<0.001
Total dairy^5^ (g/d)	512.5 (379.3)	449.9 (326.2)	<0.001
Nonfermented dairy^6^ (g/d)	298.3 (309.9)	207.7 (235.3)	<0.001
Metabolic syndrome and components			
Metabolic syndrome (%)	42.5	34.1	<0.001
Fasting glucose (mmol/L)	5.9 (1.3)	5.7 (1.1)	<0.001
Triglycerides (mmol/L)	1.5 (1.1)	1.3 (0.8)	<0.001
HDL (mmol/L)	1.4 (0.3)	1.6 (0.4)	<0.001
Mean arterial pressure (mm Hg)	100.1 (12.1)	97.4 (12.6)	<0.001
Waist circumference (cm)	99.2 (12.9)	89.4 (14)	<0.001
High waist circumference^7^ (%)	63.6	71.3	<0.001

Values are mean (SD) unless otherwise indicated.

1 Participants were categorized into sex- and birth cohort–specific educational tertiles based on self-reported total years of education, to account for secular trends in the expansion of the education system and the increases in mean education duration over time.

2 Participants who are currently smoking or who have quit <6 mo ago.

3 Sour milk, viili (Finnish fermented milk product), quark, and yogurt.

4 Aged cheese, processed cheese, and spreadable cheese.

5 All dairy products.

6 Milk.

7 ≥94 cm for men and ≥80 cm for women.

∗∗∗∗ *P* value for difference between men and women analyzed with χ^2^ for categorical variables and independent samples *t*-test for continuous variables.

### Associations between fermented dairy consumption and MetS

Fermented dairy (excluding cheese) consumption in the highest quintile (Q5) was associated with 28% lower odds (95% CI: 0.53, 0.98, *P*-trend = 0.018, model 2) of MetS in men (Table 2) when compared with the lowest quintile (Q1). In women, no statistically significant associations between fermented dairy and MetS were found when the highest quintile was compared with the lowest (Table 3). In men, a 200 g increase in fermented dairy consumption was associated with a 10% lower odds of MetS (95% CI: 0.81, 1.00). A similar dose-response association between fermented dairy consumption and MetS was not evident in women.

**TABLE 2:** Associations between MetS and consumption quintiles of different dairy product groups in 2250 men.

Dairy product group	Quintile 1 (*n =* 450)	Quintile 2 (*n =* 450)	Quintile 3 (*n =* 451)	Quintile 4 (*n =* 449)	Quintile 5 (*n =* 450)	*P-*trend∗	Dose response for 200 g of fermented, total, and nonfermented dairy and 30 g of cheese OR (95% CI)	Quadratic term *P* value∗∗
Fermented dairy, mean (g/d ± SD)	9 ± 8	38 ± 16	130 ± 27	196 ± 26	503 ± 224		—	
MetS (%)	48	43	43	43	35		—	
Model 1 OR (95% CI)	1.00 (ref)	0.99 (0.76, 1.32)	0.93 (0.71, 1.21)	0.75 (0.57, 0.99)	0.61 (0.46, 0.82)	<0.001	0.86 (0.78, 0.95)	0.4
Model 2 OR (95% CI)	1.00 (ref)	1.12 (0.84, 1.49)	1.03 (0.78, 1.37)	0.93 (0.69, 1.25)	0.72 (0.53, 0.98)	0.018	0.90 (0.81, 1.00)	0.96
Cheese, mean (g/d ± SD)	9 ± 5	26 ± 6	38 ± 6	64 ± 6	114 ± 42		—	
MetS (%)	47	43	40	43	40		—	
Model 1 OR (95% CI)	1.00 (ref)	1.16 (0.88, 1.53)	0.87 (0.65, 1.17)	0.89 (0.68, 1.19)	0.99 (0.74, 1.35)	0.09	1.02 (0.96, 1.10)	0.1
Model 2 OR (95% CI)	1.00 (ref)	1.18 (0.88, 1.58)	0.9 (0.66, 1.24)	0.86 (0.63, 1.17)	0.85 (0.61, 1.19)	0.22	0.99 (0.91, 1.07)	0.24
Total dairy, mean (g/d ± SD)	99 ± 52	265 ± 38	411 ± 70	659 ± 62	1061 ± 310		—	
MetS (%)	47	45	35	45	46		—	
Model 1 OR (95% CI)	1.00 (ref)	0.87 (0.66, 1.15)	0.64 (0.48, 0.84)	0.92 (0.69, 1.21)	0.99 (0.73, 1.33)	0.022	1.00 (0.95, 1.05)	0.012
Model 2 OR (95% CI)	1.00 (ref)	0.91 (0.68, 1.21)	0.69 (0.52, 0.92)	1.04 (0.77, 1.39)	1.09 (0.79, 1.51)	0.15	1.00 (0.94, 1.06)	0.06
Nonfermented, mean (g/d ± SD)	17 ± 27	66 ± 32	179 ± 31	450 ± 97	690 ± 266		—	
MetS (%)	47	45	43	48	49		—	
Model 1 OR (95% CI)	1.00 (ref)	0.93 (0.71, 1.22)	0.87 (0.65, 1.17)	1.12 (0.85, 1.48)	1.14 (0.86, 1.52)	0.24	1.05 (0.99, 1.11)	0.11
Model 2 OR (95% CI)	1.00 (ref)	0.94 (0.71, 1.25)	0.9 (0.66, 1.24)	1.19 (0.88, 1.59)	1.19 (0.87, 1.62)	0.48	1.05 (0.98, 1.12)	0.23

Quintile median consumptions (Q1–Q5) used for trend tests were: fermented dairy 6, 35, 126, 200, and 441 g/d; cheese 10, 26, 38, 64, and 104 g/d; total dairy 98, 267, 429, 655, and 1021 g/d; nonfermented dairy 4, 61, 203, 500, and 624 g/d.

Model 1 adjusted for age and energy intake.

Model 2 adjusted for model 1 + education, smoking, physical activity, alcohol intake, sucrose, SFAs, and fruits, berries, and vegetables consumption.

Abbreviations: CI, confidence interval; MetS, metabolic syndrome; OR, odds ratio; ref, reference group.

∗ *P* value for trend tested with linear regression represents the statistical significance of the linear trend across medians of consumption quintile.

∗∗ Quadratic term *P* value for nonlinear trend across medians of consumption quintiles.

**TABLE 3:** Associations between MetS and consumption quintiles of different dairy product groups in 2846 women.

Dairy product group	Quintile 1 (*n =* 569)	Quintile 2 (*n =* 565)	Quintile 3 (*n =* 569)	Quintile 4 (*n =* 573)	Quintile 5 (*n =* 570)	*P*-trend∗	Dose response for 200 g of fermented, total, and nonfermented dairy and 30 g of cheese OR (95% CI)	Quadratic term *P* value∗∗
Fermented dairy, mean (g/d ± SD)	10 ± 8	73 ± 22	131 ± 26	228 ± 27	483 ± 236		—	
MetS (%)	37	35	29	40	37		—	
Model 1 OR (95% CI)	1.00 (ref)	0.95 (0.72, 1.24)	0.70 (0.53, 0.94)	1.02 (0.78, 1.34)	0.83 (0.62, 1.11)	0.72	0.95 (0.87, 1.03)	0.35
Model 2 OR (95% CI)	1.00 (ref)	0.99 (0.74, 1.31)	0.72 (0.54, 0.97)	1.13 (0.85, 1.49)	0.92 (0.68, 1.25)	0.64	0.98 (0.89, 1.08)	0.51
Cheese, mean (g/d ± SD)	10 ± 5	23 ± 6	41 ± 6	55 ± 7	109 ± 42		—	
MetS (%)	41	36	32	30	35		—	
Model 1 OR (95% CI)	1.00 (ref)	0.77 (0.59, 1.01)	0.66 (0.51, 0.86)	0.54 (0.41, 0.71)	0.73 (0.55, 0.98)	0.06	0.97 (0.91, 1.05)	<0.001
Model 2 OR (95% CI)	1.00 (ref)	0.79 (0.61, 1.06)	0.72 (0.55, 0.94)	0.59 (0.43, 0.79)	0.73 (0.54, 1.00)	0.046	0.99 (0.91, 1.07)	<0.001
Total dairy, mean (g/d ± SD)	97 ± 52	247 ± 37	382 ± 48	567 ± 60	952 ± 295		—	
MetS (%)	32	33	34	36	38		—	
Model 1 OR (95% CI)	1.00 (ref)	1.04 (0.79, 1.37)	1.16 (0.89, 1.53)	1.25 (0.95, 1.64)	1.46 (1.09, 1.96)	0.62	1.04 (0.98, 1.10)	0.8
Model 2 OR (95% CI)	1.00 (ref)	1.09 (0.81, 1.44)	1.19 (0.89, 1.58)	1.33 (0.99, 1.78)	1.46 (1.07, 2.01)	0.37	1.11 (1.09, 1.14)	0.83
Nonfermented, mean (g/d ± SD)	16 ± 9	37 ± 18	165 ± 38	331 ± 89	625 ± 208		—	
MetS (%)	33	35	34	37	40		—	
Model 1 OR (95% CI)	1.00 (ref)	1.15 (0.88, 1.49)	1.10 (0.84, 1.44)	1.22 (0.94, 1.59)	1.64 (1.25, 2.14)	0.55	1.12 (1.05, 1.19)	0.62
Model 2 OR (95% CI)	1.00 (ref)	1.15 (0.87, 1.53)	1.09 (0.83, 1.45)	1.18 (0.89, 1.56)	1.44 (1.08, 1.93)	0.59	1.10 (1.03, 1.17)	0.84

Quintile median consumptions (Q1–Q5) used for trend tests were: fermented dairy 10, 78, 127, 224, and 412 g/d; cheese 11, 22, 41, 54, and 94 g/d; total dairy 99, 249, 379, 564, and 863 g/d; nonfermented dairy 3, 38, 137, 238, and 491 g/d

Model 1 adjusted for age and energy intake.

Model 2 adjusted for model 1 + education, smoking, physical activity, alcohol intake, sucrose, SFAs, and fruits, berries, and vegetables consumption.

Abbreviations: CI, confidence interval; MetS, metabolic syndrome; OR, odds ratio; ref, reference group.

∗ *P* value for trend tested with linear regression represents the statistical significance of the linear trend across medians of consumption quintiles.

∗∗ Quadratic term *P* value for nonlinear trend across medians of consumption quintiles.

For cheese, no significant trend was observed in men (model 2: *P*-trend = 0.22) (Table 2). In women, cheese consumption in the highest quintile (Q5 median: 94 g/d) was associated with a borderline significant lower odds in MetS compared with the lowest (OR: 0.73, 95% CI: 0.54, 1.00, *P*-trend = 0.046, quadratic term *P* value <0.001, model 2) quintile (Q1 median: 10.8 g/d).

### Associations between other dairy consumption and MetS

The total consumption of dairy products was associated with 31% lower odds of MetS for men only in the third quintile (Q3 median: 429 g/d) compared with the lowest quintile (Q1 median: 98 g/d) (OR: 0.69, 95% CI: 0.52, 0.92, *P*-trend = 0.15, quadratic term *P* value = 0.06, model 2) (Table 2). In women, statistically significant associations were found in the highest compared with the lowest quintile comparison (OR: 1.46, 95% CI: 1.07, 2.01, *P*-trend = 0.37, quadratic term *P* value = 0.83) and in the dose-response analysis for a 200 g increment (OR: 1.11, 95% CI: 1.09, 1.14) (Table 3).

For nonfermented dairy and MetS, no significant associations or trends were found in men (Table 2). In women, consumption of nonfermented dairy in the highest quintile (Q5 median: 491 g/d) was associated with 44% higher odds of MetS compared with the lowest quintile (Q1 median: 3 g/d) (OR: 1.44, 95% CI: 1.08, 1.93, *P*-trend = 0.59, quadratic term *P* value = 0.84 model 2). In the dose-response analysis for a 200 g increment, the odds for MetS were 10% higher (95% CI: 1.03, 1.17) in women (Table 3).

### Associations between dairy consumption and individual MetS components

Fermented dairy (excluding cheese) consumption in men was not associated with any MetS components in model 2 (Table 4). In women, TG concentrations were 0.15 mmol/L lower in the highest compared with the lowest quintile (1.20 ± 0.04 compared with 1.35 ± 0.03 mmol/L, *P*-trend = 0.06, quadratic term *P* value = 0.06, model 2) (Table 5). In dose-response analysis, each 200 g increase in fermented dairy consumption was associated with 0.03 mmol/L lower TGs (95% CI: –0.06, –0.01).

**TABLE 4:** EMMs (SE) and dose–dependent changes in MetS components across consumption quintiles of different dairy product groups in 2250 men.

Dairy product group		Quintile 1 (*n =* 450)	Quintile 2 (*n =* 450)	Quintile 3 (*n =* 451)	Quintile 4 (*n =* 449)	Quintile 5 (*n =* 450)	*P*-trend∗∗	Dose response (per 200 g for fermented/total/nonfermented dairy and per 30 g for cheese), Δ (95% CI)	Quadratic term *P* value∗∗∗
Fermented dairy, mean (g/d ± SD)		9 ± 8	38 ± 16	130 ± 27	196 ± 26	503 ± 224		—	
Fasting glucose (mmol/L)	Model 1	5.97 (0.06)	5.93 (0.06)	5.96 (0.07)	6.02 (0.07)	5.95 (0.07)	0.91	0.02 (–0.07, 0.09)	0.64
	Model 2	5.96 (0.06)	5.94 (0.06)	5.97 (0.07)	6.03 (0.07)	5.95 (0.07)	0.93	0.03 (–0.03, 0.09)	0.75
HDL (mmol/L)	Model 1	1.34 (0.01)	1.36 (0.02)	1.34 (0.02)	1.36 (0.02)	1.34 (0.02)	0.54	–0.01 (–0.02, 0.01)	0.32
	Model 2	1.34 (0.01)	1.36 (0.02)	1.34 (0.02)	1.35 (0.02)	1.34 (0.02)	0.54	–0.01 (–0.02, 0.01)	0.42
Mean arterial pressure (mm Hg)	Model 1	100.8 (0.5)	100.6 (0.6)	99.7 (0.6)	100.7 (0.6)	100.6 (0.6)	0.94	0.22 (–0.31, 0.76)	0.79
	Model 2	100.6 (0.5)	100.3 (0.6)	99.8 (0.6)	100.9 (0.6)	101.1 (0.6)	0.32	0.47 (–0.09, 1.02)	0.17
Triglycerides (mmol/L)	Model 1	1.59 (0.05)	1.51 (0.05)	1.59 (0.05)	1.52 (0.05)	1.41 (0.05)∗	0.022	–0.05 (–0.10, –0.01)	0.07
	Model 2	1.55 (0.05)	1.5 (0.05)	1.58 (0.05)	1.54 (0.05)	1.47 (0.05)	0.36	–0.03 (–0.08, 0.02)	0.35
Waist circumference (cm)	Model 1	99.6 (0.5)	98.9 (0.6)	100.1 (0.6)	98.3 (0.6)	98.7 (0.7)	0.27	–0.10 (–0.66, 0.46)	0.42
	Model 2	98.7 (0.5)	98.8 (0.6)	100.1 (0.6)	98.7 (0.6)	99.3 (0.6)	0.53	0.41 (–0.15, 0.96)	0.66
Cheese, mean (g/d ± SD)		9 ± 5	26 ± 6	38 ± 6	64 ± 6	114 ± 42		—	
Fasting glucose (mmol/L)	Model 1	6.00 (0.07)	5.93 (0.07)	6.10 (0.07)	5.92 (0.06)	5.93 (0.06)	0.36	–0.01 (–0.06, 0.03)	0.78
	Model 2	6.10 (0.07)	5.96 (0.07)	6.10 (0.07)	5.91 (0.06)	5.87 (0.07)	0.050	–0.03 (–0.08, 0.01)	0.94
HDL (mmol/L)	Model 1	1.32 (0.02)	1.33 (0.02)	1.36 (0.02)	1.37 (0.02)∗	1.36 (0.02)	0.07	0.01 (–0.01, 0.02)	0.045
	Model 2	1.33 (0.02)	1.33 (0.02)	1.35 (0.02)	1.36 (0.02)	1.36 (0.02)	0.18	0.01 (–0.01, 0.02)	0.024
Mean arterial pressure (mm Hg)	Model 1	100.0 (0.6)	101.1 (0.6)	100.5 (0.6)	100.4 (0.5)	100.5 (0.6)	0.98	0.01 (–0.39, 0.39)	0.96
	Model 2	99.9 (0.6)	101.1 (0.6)	100.6 (0.6)	100.4 (0.5)	100.5 (0.6)	0.94	–0.01 (–0.41, 0.40)	1.00
Triglycerides (mmol/L)	Model 1	1.49 (0.06)	1.57 (0.05)	1.54 (0.05)	1.51 (0.05)	1.55 (0.05)	0.82	0.01 (–0.03, 0.04)	0.78
	Model 2	1.47 (0.06)	1.58 (0.05)	1.57 (0.05)	1.52 (0.05)	1.53 (0.05)	0.97	0.01 (–0.03, 0.05)	0.98
Waist circumference (cm)	Model 1	98.4 (0.6)	99.3 (0.6)	99.6 (0.6)	98.8 (0.5)	99.7 (0.6)	0.31	0.16 (–0.25, 0.57)	0.30
	Model 2	98.4 (0.6)	99.2 (0.6)	99.6 (0.6)	98.8 (0.5)	99.4 (0.6)	0.55	0.09 (–0.32, 0.51)	0.63
Total dairy, mean (g/d ± SD)		99 ± 52	265 ± 38	411 ± 70	659 ± 62	1061 ± 310		—	
Fasting glucose (mmol/L)	Model 1	6.06 (0.07)	5.92 (0.07)	5.89 (0.07)	5.93 (0.06)	6.02 (0.06)	0.68	0.02 (–0.02, 0.06)	0.48
	Model 2	6.05 (0.07)	5.92 (0.07)	5.89 (0.07)	5.92 (0.06)	6.04 (0.06)	0.87	0.01 (–0.03, 0.05)	0.49
HDL (mmol/L)	Model 1	1.38 (0.02)	1.38 (0.02)	1.39 (0.02)	1.33 (0.02)∗	1.3 (0.02)∗	<0.001	–0.02 (–0.04, –0.02)	<0.001
	Model 2	1.36 (0.02)	1.37 (0.02)	1.37 (0.02)	1.33 (0.02)	1.32 (0.02)	0.025	–0.01 (–0.02, 0.01)	0.041
Mean arterial pressure (mm Hg)	Model 1	101.8 (0.6)	100.9 (0.6)	100.1 (0.6)∗	99.8 (0.5)∗	100.1 (0.6)∗	0.022	–0.25 (–0.55, 0.08)	0.07
	Model 2	101.5 (0.6)	100.9 (0.6)	100.1 (0.6)	100 (0.5)	100.3 (0.6)	0.08	–0.24 (–0.56, 0.11)	0.40
Triglycerides (mmol/L)	Model 1	1.53 (0.05)	1.49 (0.05)	1.48 (0.05)	1.58 (0.05)	1.55 (0.05)	0.62	0.01 (–0.02, 0.04)	0.45
	Model 2	1.5 (0.05)	1.48 (0.05)	1.48 (0.05)	1.59 (0.05)	1.58 (0.05)	0.3	–0.01 (–0.04, 0.04)	0.36
Waist circumference (cm)	Model 1	99.5 (0.6)	98.9 (0.6)	98.1 (0.6)	98.5 (0.6)	100.4 (0.6)	0.07	0.22 (–0.07, 0.56)	0.026
	Model 2	98.9 (0.6)	98.8 (0.6)	98 (0.6)	98.6 (0.6)	100.6 (0.6)	0.1	0.43 (0.11, 0.74)	0.40
Nonfermented dairy, mean (g/d ± SD)		17 ± 27	66 ± 32	179 ± 31	450 ± 97	690 ± 266		—	
Fasting glucose (mmol/L)	Model 1	5.86 (0.07)	6.17 (0.07)∗	5.87 (0.07)	5.87 (0.07)	6.02 (0.05)	0.78	0.01 (–0.03, 0.05)	0.59
	Model 2	5.84 (0.07)	6.15 (0.07)∗	5.88 (0.07)	5.86 (0.06)	6.04 (0.05)∗	0.65	0.01 (–0.03, 0.05)	0.58
HDL (mmol/L)	Model 1	1.38 (0.02)	1.38 (0.02)	1.37 (0.02)	1.35 (0.02)	1.3 (0.02)∗	<0.001	–0.02 (–0.03, –0.01)	<0.001
	Model 2	1.36 (0.02)	1.38 (0.02)	1.36 (0.02)	1.35 (0.02)	1.31 (0.01)	0.009	–0.01 (–0.02, 0.01)	0.011
Mean arterial pressure (mm Hg)	Model 1	102.5 (0.6)	100.9 (0.6)	100.2 (0.6)∗	100 (0.6)∗	99.6 (0.5)∗	<0.001	–0.42 (–0.75, –0.04)	0.008
	Model 2	102.4 (0.6)	101.1 (0.6)	100.3 (0.6)∗	99.9 (0.5)∗	99.6 (0.5)∗	<0.001	–0.52 (–0.83, –0.11)	0.015
Triglycerides (mmol/L)	Model 1	1.45 (0.06)	1.55 (0.05)	1.47 (0.05)	1.51 (0.06)	1.6 (0.04)∗	0.09	0.03 (0.01, 0.06)	0.10
	Model 2	1.45 (0.06)	1.55 (0.05)	1.46 (0.05)	1.51 (0.06)	1.61 (0.04)∗	0.27	0.01 (–0.02, 0.04)	0.22
Waist circumference (cm)	Model 1	99.5 (0.7)	99.1 (0.6)	98.3 (0.6)	97.8 (0.5)	100.2 (0.6)	0.28	0.33 (–0.09, 0.65)	0.11
	Model 2	99 (0.6)	98.9 (0.6)	98.5 (0.6)	97.8 (0.6)	100.1 (0.5)	0.57	0.37 (–0.01, 0.76)	0.45

Quintile median consumptions (Q1–Q5) used for trend tests were: fermented dairy 6, 35, 126, 200, and 441 g/d; cheese 10, 26, 38, 64, and 104 g/d; total dairy 98, 267, 429, 655, and 1021 g/d; nonfermented dairy 4, 61, 203, 500, and 624 g/d.

Values are EMMs with SEs.

Model 1 adjusted for age and energy intake.

Model 2 adjusted for model 1 + education, smoking, physical activity, alcohol intake, sucrose, SFAs, and fruits, berries, and vegetables consumption.

Abbreviations: ANCOVA, analysis of covariance; CI, confidence interval; Δ, absolute change; EMM, estimated marginal mean; MetS, metabolic syndrome.

∗ Statistically significant (*P* < 0.05) difference when compared with quintile 1 in ANCOVA post hoc analyses.

∗∗ *P* value for trend tested with linear regression represents the statistical significance of the linear trend across medians of consumption quintiles.

∗∗∗ Quadratic term *P* value for nonlinear trend across medians of consumption quintiles.

**TABLE 5:** EMMs (SE) and dose-dependent changes in MetS components across consumption quintiles of different dairy product groups in 2846 women.

Dairy product group	Quintile 1 (*n =* 569)	Quintile 2 (*n =* 565)	Quintile 3 (*n =* 569)	Quintile 4 (*n =* 573)	Quintile 5 (*n =* 570)	*P*-trend∗∗	Dose response (per 200 g for fermented/total/nonfermented dairy and per 30 g for cheese), Δ (95% CI)	Quadratic term *P* value∗∗∗
Fermented dairy, mean (g/d ± SD)	10 ± 8	73 ± 22	131 ± 26	228 ± 27	483 ± 236		—	
Fasting glucose (mmol/L)								
Model 1	5.68 (0.05)	5.68 (0.05)	5.62 (0.04)	5.64 (0.05)	5.7 (0.05)	0.99	–0.01 (–0.04, 0.04)	0.34
Model 2	5.65 (0.05)	5.66 (0.05)	5.61 (0.04)	5.61 (0.04)	5.66 (0.04)	0.71	0.02 (–0.03, 0.06)	0.16
HDL (mmol/L)								
Model 1	1.61 (0.02)	1.62 (0.02)	1.64 (0.02)	1.64 (0.02)	1.63 (0.02)∗	0.66	0.01 (–0.01, 0.02)	0.90
Model 2	1.62 (0.02)	1.62 (0.02)	1.64 (0.02)	1.63 (0.02)	1.63 (0.02)	0.79	0.01 (–0.01, 0.02)	0.96
Mean arterial pressure (mm Hg)								
Model 1	96.9 (0.6)	97.8 (0.5)	97.3 (0.5)	97.5 (0.5)	97.6 (0.5)	0.71	–0.19 (–0.65, 0.28)	0.86
Model 2	96.8 (0.6)	97.8 (0.5)	97.4 (0.5)	97.5 (0.5)	97.4 (0.5)	0.97	–0.20 (–0.69, 0.29)	0.71
Triglycerides (mmol/L)								
Model 1	1.38 (0.04)	1.25 (0.03)∗	1.22 (0.03)∗	1.27 (0.03)∗	1.18 (0.04)∗	0.003	–0.04 (–0.07, –0.01)	0.025
Model 2	1.35 (0.03)	1.22 (0.03)∗	1.21 (0.03)∗	1.27 (0.03)	1.2 (0.04)∗	0.06	–0.03 (–0.06, –0.01)	0.06
Waist circumference (cm)								
Model 1	90.6 (0.7)	89.7 (0.6)	89.2 (0.6)	89 (0.6)	88.8 (0.6)∗	0.07	–0.56 (–1.09, –0.04)	0.23
Model 2	90.1 (0.6)	89.2 (0.6)	89 (0.5)	88.9 (0.6)	89.3 (0.7)	0.77	–0.18 (–0.72, 0.36)	0.91
Cheese, mean (g/d ± SD)	10 ± 5	23 ± 6	41 ± 6	55 ± 7	109 ± 42		—	
Fasting glucose (mmol/L)								
Model 1	5.75 (0.04)	5.64 (0.04)	5.68 (0.04)	5.55 (0.05)∗	5.62 (0.05)∗	0.06	–0.02 (–0.05, 0.02)	0.15
Model 2	5.72 (0.05)	5.63 (0.04)	5.66 (0.04)	5.55 (0.05)∗	5.61 (0.05)	0.16	–0.01 (–0.04, 0.03)	0.24
HDL (mmol/L)								
Model 1	1.56 (0.02)	1.62 (0.02)∗	1.64 (0.02)∗	1.65 (0.02)∗	1.68 (0.02)∗	<0.001	0.02 (0.01, 0.03)	<0.001
Model 2	1.58 (0.02)	1.62 (0.02)	1.64 (0.02)∗	1.64 (0.02)∗	1.67 (0.02)∗	<0.001	0.01 (0.01, 0.03)	<0.001
Mean arterial pressure (mm Hg)								
Model 1	97.5 (0.5)	98.1 (0.5)	96.9 (0.5)	97.5 (0.6)	97.3 (0.5)	0.61	–0.01 (–0.38, 0.36)	0.5
Model 2	97.3 (0.5)	97.9 (0.5)	97 (0.5)	97.7 (0.6)	97.1 (0.6)	0.63	–0.06 (–0.45, 0.33)	0.25
Triglycerides (mmol/L)								
Model 1	1.35 (0.03)	1.26 (0.03)∗	1.21 (0.03)∗	1.23 (0.03)∗	1.21 (0.04)∗	0.008	–0.03 (–0.05, –0.01)	0.021
Model 2	1.34 (0.03)	1.25 (0.03)∗	1.21 (0.03)∗	1.23 (0.03)∗	1.2 (0.03)∗	0.020	–0.02 (–0.05, 0.01)	0.07
Waist circumference (cm)								
Model 1	90.6 (0.7)	89.4 (0.6)	88.5 (0.5)∗	88.4 (0.6)∗	89.7 (0.6)	0.41	0.18 (–0.24, 0.60)	0.84
Model 2	89.9 (0.6)	89.3 (0.6)	88.8 (0.5)	88.5 (0.6)	89.8 (0.6)	0.91	0.40 (–0.03, 0.83)	0.98
Total dairy, mean (g/d ± SD)	97 ± 52	247 ± 37	382 ± 48	567 ± 60	952 ± 295		—	
Fasting glucose (mmol/L)								
Model 1	5.67 (0.05)	5.64 (0.04)	5.63 (0.04)	5.64 (0.05)	5.7 (0.05)	0.64	0.01 (–0.02, 0.03)	0.41
Model 2	5.65 (0.05)	5.62 (0.04)	5.61 (0.04)	5.63 (0.05)	5.68 (0.05)	0.86	0.02 (–0.01, 0.05)	0.69
HDL (mmol/L)								
Model 1	1.65 (0.02)	1.64 (0.02)	1.63 (0.02)	1.63 (0.02)	1.58 (0.02)∗	0.015	–0.01 (–0.02, –0.01)	0.004
Model 2	1.64 (0.02)	1.63 (0.02)	1.62 (0.02)	1.64 (0.02)	1.61 (0.02)∗	0.65	–0.01 (–0.02, 0.01)	0.34
Mean arterial pressure (mm Hg)								
Model 1	98.4 (0.5)	97.3 (0.5)	97.8 (0.5)	96.8 (0.5)∗	96.7 (0.6)∗	0.06	–0.4 (–0.70, –0.01)	0.06
Model 2	98.3 (0.5)	97.3 (0.5)	97.8 (0.5)	96.8 (0.5)	96.6 (0.6)	0.08	–0.31 (–0.71, 0.05)	0.08
Triglycerides (mmol/L)								
Model 1	1.28 (0.03)	1.23 (0.03)	1.26 (0.03)	1.23 (0.03)	1.26 (0.04)	0.60	–0.01 (–0.03, 0.01)	0.91
Model 2	1.27 (0.03)	1.22 (0.03)	1.26 (0.03)	1.23 (0.03)	1.25 (0.04)	0.25	–0.01 (–0.03, 0.01)	0.59
Waist circumference (cm)								
Model 1	88.6 (0.6)	89.3 (0.6)	89.2 (0.5)	89.3 (0.6)	90.6 (0.7)∗	0.05	0.32 (–0.04, 0.62)	0.035
Model 2	88.3 (0.6)	89.2 (0.6)	89.3 (0.5)	89.5 (0.6)	90.3 (0.7)∗	0.29	0.41 (–0.08, 0.79)	0.13
Nonfermented dairy, mean (g/d ± SD)	16 ± 9	37 ± 18	165 ± 38	331 ± 89	625 ± 208		—	
Fasting glucose (mmol/L)								
Model 1	5.58 (0.04)	5.66 (0.04)	5.7 (0.04)∗	5.65 (0.04)	5.71 (0.06)	0.09	0.02 (–0.02, 0.05)	0.25
Model 2	5.58 (0.04)	5.65 (0.04)	5.68 (0.04)	5.63 (0.04)	5.68 (0.06)	0.54	0.02 (–0.02, 0.04)	0.87
HDL (mmol/L)								
Model 1	1.67 (0.02)	1.63 (0.02)	1.64 (0.02)	1.61 (0.02)∗	1.55 (0.02)∗	<0.001	–0.03 (–0.06, –0.02)	<0.001
Model 2	1.66 (0.02)	1.63 (0.02)	1.63 (0.02)∗	1.62 (0.02)∗	1.58 (0.02)∗	0.08	–0.02 (–0.03, –0.01)	0.009
Mean arterial pressure (mm Hg)								
Model 1	97.9 (0.5)	98.3 (0.5)	96.9 (0.5)	97.2 (0.5)	96.5 (0.7)	0.11	–0.44 (–0.89, 0.05)	0.14
Model 2	97.7 (0.5)	98.2 (0.5)	96.9 (0.5)	97.3 (0.5)	96.6 (0.7)	0.21	–0.39 (–0.82, 0.01)	0.35
Triglycerides (mmol/L)								
Model 1	1.21 (0.03)	1.28 (0.03)∗	1.22 (0.03)∗	1.27 (0.03)	1.33 (0.04)∗	0.06	0.02 (–0.04, 0.04)	0.020
Model 2	1.22 (0.03)	1.27 (0.03)	1.22 (0.03)	1.25 (0.03)	1.29 (0.04)	0.75	0.03 (–0.02, 0.04)	0.35
Waist circumference (cm)								
Model 1	88.1 (0.5)	89 (0.5)	89.1 (0.5)	90.2 (0.5)∗	91.6 (0.8)∗	<0.001	0.84 (0.41, 1.29)	<0.001
Model 2	88.3 (0.5)	88 (0.5)	89.1 (0.5)	89.9 (0.6)∗	90.7 (0.8)∗	0.16	0.54 (–0.01, 1.02)	0.12

Quintile median consumptions (Q1–Q5) used for trend tests were: fermented dairy 10, 78, 127, 224, and 412 g/d; cheese 11, 22, 41, 54, and 94 g/d; total dairy 99, 249, 379, 564, and 863 g/d; nonfermented dairy 3, 38, 137, 238, and 491 g/d.

Values are EMMs with SEs.

Model 1 adjusted for age and energy intake.

Model 2 adjusted for model 1 + education, smoking, physical activity, alcohol intake, sucrose, SFAs, and fruits, berries, and vegetables consumption.

Abbreviations: ANCOVA, analysis of covariance; CI, confidence interval; Δ, absolute change; EMM, estimated marginal mean; MetS, metabolic syndrome.

∗ Statistically significant (*P* < 0.05) difference when compared with quintile 1 in ANCOVA post hoc analyses.

∗∗ *P* value for trend tested with linear regression represents the statistical significance of the linear trend across medians of consumption quintiles.

∗∗∗ Quadratic term *P* value for nonlinear trend across medians of consumption quintiles.

Cheese consumption in men was not associated with any MetS components in model 2, except for a borderline significant declining trend in fasting glucose concentrations (*P*-trend = 0.050) and a significant nonlinear trend (quadratic term *P* value = 0.024) (Table 4). In women, cheese consumption was associated with higher HDL cholesterol and lower TG concentrations. HDL cholesterol was 0.09 mmol/L higher in the highest compared with the lowest quintile (1.67 ± 0.02 mmol/L compared with 1.58 ± 0.02 mmol/L, *P*-trend ≤0.001, quadratic term *P* value ≤0.001), and TGs were 0.14 mmol/L lower (1.20 ± 0.03 mmol/L compared with 1.34 ± 0.03 mmol/L, *P*-trend = 0.020, quadratic term *P* value = 0.07) (Table 5).

Total dairy consumption in men was associated with lower HDL cholesterol (Table 4). HDL cholesterol was 0.04 mmol/L lower in the highest compared with the lowest quintile (1.32 ± 0.02 mmol/L compared with 1.36 ± 0.02 mmol/L, *P*-trend = 0.025, quadratic term *P* value = 0.041, model 2). In dose-response analysis, waist circumference was 0.43 cm higher for each 200 g increment (95% CI: 0.11, 0.74). In women, total dairy consumption was not associated with MetS components in model 2 (Table 5).

Higher nonfermented dairy consumption in men was associated with higher TGs, lower HDL cholesterol, and lower MAP. TGs were 0.16 mmol/L higher in the highest compared with the lowest quintile (1.61 ± 0.04 mmol/L compared with 1.45 ± 0.06 mmol/L, *P*-trend = 0.27, quadratic term *P* value = 0.22, model 2), HDL cholesterol was 0.05 mmol/L lower (1.31 ± 0.01 mmol/L compared with 1.36 ± 0.02 mmol/L, *P*-trend = 0.009, quadratic term *P* value = 0.011, model 2), and MAP was 2.8 mmHg lower (99.6 ± 0.5 mmHg compared with 102.4 ± 0.6 mmHg, *P*-trend ≤0.001, quadratic term *P* value = 0.015, model 2) in lowest compared with highest quintile comparisons (Table 4). In women, nonfermented dairy consumption was associated with 0.08 mmol/L lower HDL concentrations (1.66 ± 0.02 compared with 1.50 ± 0.02 mmol/L, *P*-trend = 0.08, quadratic term *P* value = 0.009, model 2) and 2.4 cm higher waist circumference (88.3 ± 0.5 compared with 90.7 ± 0.8 cm, *P*-trend = 0.16, quadratic term *P* value = 0.12, model 2).

Sensitivity analyses excluding participants on antihypertensive, diabetes, or lipid-lowering medication, and models adjusting for lipid-lowering medication, yielded similar results (data not shown). In a sensitivity analysis separating high- and low-fat cheese, no statistically significant linear or nonlinear trends in MetS were observed in either men or women after full adjustment (Supplementary Tables 2 and 3).

## Discussion

In this large, population-based Finnish cross-sectional study, higher consumption of fermented dairy products (excluding cheese) was associated with a lower prevalence of MetS in men, whereas in women, fermented dairy was associated with lower TG concentrations but not with MetS overall. Cheese showed an inverse association with MetS in women, accompanied by higher HDL cholesterol and lower TGs. In men, cheese consumption was not clearly associated with MetS, though a borderline inverse association with fasting glucose was observed. Higher nonfermented dairy (milk) consumption was associated with a higher prevalence of MetS, especially in women, and associated with lower HDL cholesterol levels in both sexes. For total dairy consumption, higher consumption was associated with a higher prevalence of MetS in women. These findings suggest that associations between dairy consumption and metabolic health may differ by both sex and dairy subgroup.

This is the first study, to our knowledge, to examine also the nonlinear (quadratic) associations between dairy consumption and MetS components separately by sex. Among women, a nonlinear association was observed for cheese, with mid- to high consumption associated with lower MetS prevalence and higher HDL, suggesting the association may not increase proportionally across the consumption range. In men, no nonlinear pattern was evident for MetS overall, although HDL and blood pressure showed opposite curvature with nonfermented milk, which may help explain the absence of a clear pattern at the syndrome level. Together, these patterns suggest that sex-specific associations may be driven primarily by differences in lipid-related components and by subtype-specific consumption response shapes, such that opposing component-level curvature may attenuate any overall association with MetS in men.

Only 2 previous studies provide comparable context for our findings: Korean Genome and Epidemiology Study (KoGES) and Suivi Temporaire Annuel Non-Invasif de la Santé des Lorrains Assurés Sociaux (STANISLAS), both of which highlight potential sex-specific differences in dairy consumption and MetS [16,17]. KoGES included 5510 (2859 men and 2651 women) aged 40–69 y, with an mean follow-up of 67.4 mo, and evaluated milk, yogurt, and total dairy intake in relation to MetS, stratified by sex [17]. Higher yogurt (fermented dairy) intake was associated with lower overall MetS incidence, with inverse associations across several components; HDL improvement was significant only in women. KoGES also reported robust inverse associations between total dairy and MetS, and favorable component patterns [17].

We likewise observed a protective association with fermented dairy (excluding cheese), but this was most evident in men. In women, fermented dairy was not associated with MetS overall, but higher consumption was modestly associated with lower TGs. In contrast to KoGES, our total dairy results were not uniformly protective: in men, benefits were limited to moderate consumption; in women, higher total dairy was associated with higher MetS odds. Component analyses suggested that higher total dairy was associated with lower HDL and larger waist circumference in men, diverging from KoGES’s generally favorable pattern. In contrast, in women, total dairy was associated with lower MAP, consistent with protective signals in KoGES [17].

STANISLAS enrolled 288 men and 300 women (28–60 y) with 5 y of follow-up, relating total dairy, milk/yogurt/cottage cheese, and cheese to changes in MetS components and formally testing sex interactions [16]. Greater milk/yogurt/cottage cheese intake was associated with more favorable 5-y glucose and HDL profiles in men; in women, higher baseline total dairy intake was associated with less favorable lipid profiles [16].

Our component-level findings in men were broadly consistent with those reported in STANISLAS. For total dairy, the higher MetS odds and less favorable waist circumference and HDL cholesterol profiles observed in women were more similar in direction to STANISLAS than to KoGES, suggesting that dairy MetS associations vary by population context (e.g., background diet, dairy types consumed, and correlated lifestyle factors) [16,17]. For nonfermented dairy, we observed nonbeneficial associations in women, especially with lower HDL and higher waist circumference. In men, nonfermented dairy was associated with lower HDL and higher TGs, but showed a modest favorable signal for MAP. These patterns diverge from KoGES’s generally protective findings. Still, they are more consistent with the sex-dependent, mixed results seen in STANISLAS, potentially reflecting differences in consumption distributions, population, product matrices (e.g., fat content, fermentation, and processing), and baseline metabolic risk.

Across KoGES, STANISLAS, and our study, fermented dairy emerges as the most consistently favorable category for MetS risk or related components, with clearer signals in men and component-specific signals (e.g., TGs, HDL) in women. The divergence from KoGES for total and nonfermented dairy, alongside similarity to STANISLAS in females’s lipid and waist patterns, underscores the importance of population context and product type. It is important to note that the KoGES population was limited to middle-aged and older people, and the 2 highest quintiles consumed approximately the same amount of dairy products per week as the highest quintile of our population did in a day. The STANISLAS population consumed an average of 2–3 servings of dairy products per day, which is less than the average consumption in our study. In addition, the range of dairy product consumption and the sample size were smaller than in our study. These differences in population and dairy product consumption may partly explain the variation in the results.

Controlled trials give an insight into the possible causality behind the associations. A meta-analysis of 52 randomized controlled trials (RCTs) examining probiotic-supplemented fermented dairy products (e.g., yogurt, kefir, and cheese) and capsule/powder formulations revealed significant improvements in MetS–related factors [10]. In trials using a dairy matrix, fasting glucose decreased by a weighted mean difference of –0.37 mmol/L, HDL cholesterol rose by +0.26 mmol/L, and triacylglycerols declined by –0.46 mmol/L, often involving strains such as *Lactobacillus helveticus, L. casei, L. acidophilus,* and *Bifidobacterium lactis*. Studies using probiotic capsules or powders also reported modest but notable reductions in body weight (–0.26 kg) and BMI (–0.35 kg/m^2^) [10]. Although these findings align with ours, particularly regarding the favorable effects of fermented dairy consumption on lipid biomarkers, the potential differences by sex remain unobserved.

Fermented dairy consumption may influence metabolic health via several interlinked mechanisms. Gut microbial fermentation of these products increases populations of beneficial bacteria, which generate short-chain fatty acids (SCFAs: acetate, propionate, and butyrate). SCFAs can improve lipid metabolism (reducing cholesterol synthesis and TGs), activate anti-inflammatory pathways, and enhance glucose regulation [[28], [29], [30], [31]]. Bioactive peptides formed during fermentation (e.g., valine-proline-proline, isoleucyl-proline-proline) may inhibit angiotensin-I-converting enzyme, lower blood pressure, and support insulin sensitivity; dairy calcium may additionally aid insulin secretion [[29], [30], [31], [32]]. Beneficial bacteria such as *Lactobacillus acidophilus* and *Bifidobacterium longum* may further support cholesterol metabolism via bile salt deconjugation [31].

There are also a few sex-specific differences in hormonal, metabolic, and microbial mechanisms that may modulate these effects. Estrogens (notably 17β-estradiol) enhance hepatic lipid clearance, reduce lipogenesis, and increase insulin sensitivity via estrogen receptor actions in liver and adipose tissue; these effects are more marked in premenopausal women and contribute to better lipid profiles, fat distribution, and metabolic flexibility relative to men [33,34]. Sex differences in gut microbiota composition and associated bile acid pathways may further shape lipid-metabolism gene expression and metabolic responses [[35], [36], [37]], offering a possible mechanistic explanation for the sex-specific associations observed in our study.

Although saturated fat intake has been proposed as a risk factor for metabolic disorders, especially for unfavorable changes in blood lipids, the evidence for this association is unclear. Dairy-derived saturated fat, particularly from fermented products, has been studied in a controlled trial of 49 adults, which found that saturated fat from cheese did not have an adverse effect on blood lipids, unlike the same amount from butter, highlighting the importance of the dairy matrix [38]. Similarly, a 12-wk RCT on 139 subjects found no adverse effects on metabolic markers when participants consumed high-fat cheddar cheese compared with reduced-fat cheese [39]. Previous studies also support a neutral or even protective role for full-fat fermented dairy, for instance, an Australian cohort of 1529 men and women found that the positive association with dairy did not differ between high- and low-fat dairy consumption [40]. This aligns with our findings from investigating the risk of MetS between high- and low-fat cheeses, without finding any significant differences. Collectively, these findings support the view that the metabolic effects of saturated fat may depend on the food matrix and that saturated fat from fermented dairy may not be associated with adverse metabolic effects.

This study has multiple strengths: it is population-based, with a large sample; includes detailed medical examinations that provide measured MetS variables; and investigates dairy consumption with a well-established FFQ [23,24]. We had broad variability in dairy consumption, which enhances the power to detect associations. Comprehensive adjustment was made for many potential confounders (including education, smoking, physical activity, alcohol, energy intake, saturated fat, and fruit/vegetable consumption). Despite careful adjustments, it should be noted that, on average, men in our data have less healthy lifestyles: more alcohol consumption and smoking, less consumption of vegetables, berries, and fruit, and more added sugar and saturated fat, which may affect the analyses.

Also, some limitations remain. Dietary intake was assessed by an FFQ, a widely used and validated tool, but 1 subject to recall error, misreporting, and misclassification of usual intake. Information on the use of antihypertensive and diabetes medication was self-reported, which may have introduced some degree of misclassification. However, as such medications are typically used on a regular basis and are well recognized by users, any potential misreporting is likely to be limited and would most likely bias the results toward the null. In Finnish validation studies, FFQs have sometimes overestimated or underestimated nutrient or food group consumption compared with detailed dietary records, particularly for high-fat dairy products [23,24,41]. Such measurement error may attenuate true associations toward the null, or cause bias in either direction. We adjusted for total energy intake and excluded implausible energy reporters, which helps reduce but does not eliminate bias. We were not able to distinguish SFAs derived from dairy compared with other dietary sources, limiting mechanistic inference on the potential role of different food matrices, although we adjusted for total SFA intake. The cross-sectional design precludes causal inference and raises the possibility of reverse causation; residual confounding from unmeasured or imperfectly measured factors may persist. Finally, generalizability outside similar Finnish adult populations may be limited.

In conclusion, associations between dairy subtypes and MetS appeared to differ by sex: fermented dairy (excluding cheese) was inversely associated with MetS in men, whereas cheese showed more favorable associations with MetS and lipid profiles in women. In contrast, nonfermented and total dairy showed mixed, generally less favorable associations, particularly in women. These subtype-specific patterns may reflect differences in the dairy food matrix, including fermentation-related components, but causal inference is limited by the cross-sectional design. Prospective studies and trials are needed to clarify directionality and the potential role of fermentation and food matrix in the observed sex differences.

## Author contributions

The authors’ responsibilities were as follows – MW-E: had full access to the data, analyzed the data, and drafted the manuscript; MW-E, MM, TT: had primary responsibility for the final content; and all authors: designed and conducted research, read and approved the final manuscript.

## Data availability

The dataset used is available upon request through the Findata permit procedure. https://www.findata.fi/en/.

## Funding

Valio Ltd partially funded MWE. Valio Ltd had no role in the design of the study; collection, analysis, or interpretation of data; writing of the report; or decision to submit the manuscript for publication.

## Conflict of interest

TT is employed by Valio Ltd, which also provided partial funding for the study. MWE received funding from Valio Ltd. All other authors report no conflicts of interest.
