# Supplementary material for: Fermented Dairy Consumption and Metabolic Syndrome in Finnish Men and Women
Source: J Nutr. 2026 Mar 2;156(5):101464. doi: 10.1016/j.tjnut.2026.101464 (PMC13197939; doi:10.1016/j.tjnut.2026.101464)
Supplement: Multimedia component 1 [file mmc1.docx]

Fermented Dairy Consumption and Metabolic Syndrome in Finnish Men and Women

Miika M. Wynne-Ellis et al.

**Supplementary material**

Supplementary Figure 1. Flow diagram of the National FinHealth 2017 sample. Abbreviations: FFQ, food frequency questionnaire; n, number of participants.

| Supplementary Table 1. Mean consumption (g/d ± SD) for dairy products included in fermented, non-fermented, and cheese dairy groups. | | | | | | |
| --- | --- | --- | --- | --- | --- | --- |
|  | MetS (n=1,927) | | No MetS (n=3,169) | | Total  (n=5,096) | |
|  | Mean | SD | Mean | SD | Mean | SD |
| **Total dairy (fermented + non-fermented)** | 552.9 | 408 | 538.5 | 395.8 | 543.9 | 400.5 |
| **Fermented dairy** | 220.6 | 279.4 | 224.6 | 274.3 | 223.1 | 276.2 |
| Yogurt (>1% fat) | 61.4 | 96.8 | 65.6 | 100.7 | 64.0 | 99.2 |
| Yogurt (<=1% fat) | 26.3 | 58.5 | 25.0 | 58.7 | 25.5 | 58.6 |
| Viili (Finnish fermented milk product) | 7.5 | 10.2 | 7.7 | 10.4 | 7.7 | 10.4 |
| Quark | 28.5 | 62.2 | 47.6 | 100.5 | 40.4 | 88.5 |
| Sour milk | 48.4 | 111.7 | 39.3 | 100.8 | 42.7 | 105.1 |
| **Non-fermented dairy** | 284.5 | 283.4 | 264.2 | 271.2 | 271.9 | 276.0 |
| Low-fat milk (<2% fat) | 136.0 | 206.2 | 130.2 | 201.8 | 132.4 | 203.4 |
| High-fat milk (>2% fat) | 18.5 | 84.5 | 15.2 | 77.2 | 16.4 | 80.0 |
| Skimmed milk | 105.8 | 204.5 | 95.1 | 188.4 | 99.1 | 194.7 |
| **Cheese** | 47.8 | 42.5 | 49.7 | 39.0 | 49.0 | 40.3 |
| Cheese aged (>17% fat) | 20.4 | 22.0 | 19.5 | 20.2 | 19.9 | 20.9 |
| Cheese aged (<=17% fat) | 8.8 | 15.3 | 9.2 | 15.6 | 9.0 | 15.5 |
| Processed cheese (>17% fat) | 1.8 | 3.2 | 1.5 | 2.4 | 1.6 | 2.7 |
| Processed cheese (<=17% fat) | 0.5 | 1.4 | 0.4 | 1.0 | 0.4 | 1.2 |
| Cream cheese (>15% fat) | 5.1 | 7.9 | 5.3 | 9.0 | 5.2 | 8.6 |
| Cream cheese (<=15% fat) | 11.2 | 26.2 | 13.8 | 26.7 | 12.8 | 26.6 |
| Abbreviations: g/d, grams per day; MetS, metabolic syndrome; SD, standard deviation. | | | | | | |

| Supplementary Table 2. Associations between metabolic syndrome (MetS) and consumption quintiles of high and low-fat cheese in 2,250 men. | | | | | | | |
| --- | --- | --- | --- | --- | --- | --- | --- |
|  | Quintile 1 (n=450) | Quintile 2 (n=450) | Quintile 3 (n=451) | Quintile 4 (n=449) | Quintile 5 (n=450) | p-value for trend* | Quadratic term p-value** |
| High-fat cheese, median (g/d ± SD) | 4.3±2.1 | 12.8±3.0 | 23.4±2.8 | 42.1±9.9 | 74.8±24.5 |  |  |
| Model 1 | 1.00 (ref) | 1.27 (0.97-1.68) | 1.51 (1.14-1.99) | 1.34 (1.01-1.76) | 1.54 (1.15-2.07) | 0.052 | 0.168 |
| Model 2 | 1.00 (ref) | 1.23 (0.95-1.72) | 1.54 (1.14-2.07) | 1.19 (0.87-1.62) | 1.22 (0.85-1.73) | 0.07 | 0.07 |
| Low-fat cheese, median (g/d ± SD) | 0.1±0.2 | 2.8±1.4 | 9.4±2.5 | 22.2±6.8 | 70.3±39.3 |  |  |
| Model 1 | 1.00 (ref) | 1.28 (0.97-1.68) | 1.24 (0.94-1.63) | 0.91 (0.68-1.19) | 0.93 (0.71-1.24) | 0.23 | 0.42 |
| Model 2 | 1.00 (ref) | 1.26 (0.94-1.68) | 1.31 (0.98-1.75) | 0.95 (0.71-1.28) | 1.05 (0.78-1.42) | 0.45 | 0.56 |
| Values are ORs (95% CIs). | | | | | | | |
| Model 1 adjusted for age and energy intake. | | | | | | | |
| Model 2 adjusted for model 1 + education, smoking, physical activity, alcohol intake, sucrose, and fruits, berries, and vegetables consumption. | | | | | | | |
| * p-value for trend tested with linear regression represents the statistical significance of the linear trend across medians of consumption quintiles. | | | | | | | |
| ** Quadratic term p-value for trend across medians of consumption quintiles. | | | | | | | |
| Abbreviations: CI, confidence interval; g/d, grams per day; MetS, metabolic syndrome; OR, odds ratio; ref, reference group; SD, standard deviation. | | | | | | | |

| Supplementary Table 3. Associations between metabolic syndrome (MetS) and consumption quintiles of high and low-fat cheese in 2,846 women. | | | | | | | |
| --- | --- | --- | --- | --- | --- | --- | --- |
|  | Quintile 1 (n=569) | Quintile 2 (n=565) | Quintile 3 (n=569) | Quintile 4 (n=573) | Quintile 5 (n=570) | p-value for trend* | Quadratic term p-value** |
| High-fat cheese, median (g/d ± SD) | 3.9±1.8 | 9.9±1.9 | 17.0±2.1 | 28.4±6.5 | 55.3±18.0 |  |  |
| Model1 | 1.00 (ref) | 1.07 (0.82-1.39) | 1.09 (0.84-1.43) | 0.92 (0.71-1.21) | 1.04 (0.79-1.38) | 0.64 | 0.69 |
| Model2 | 1.00 (ref) | 1.02 (0.77-1.35) | 1.09 (0.82-1.44) | 0.89 (0.67-1.21) | 0.96 (0.69-1.34) | 0.79 | 0.94 |
| Low-fat cheese, median (g/d ± SD) | 0.7±0.6 | 4.4±1.5 | 11.5±2.6 | 27.9±7.6 | 72.7±39.1 |  |  |
| Model1 | 1.00 (ref) | 1.34 (1.02-1.76) | 1.27 (0.97-1.66) | 0.87 (0.66-1.15) | 0.91 (0.69-1.19) | 0.15 | 0.39 |
| Model2 | 1.00 (ref) | 1.29 (0.97-1.73) | 1.23 (0.95-1.68) | 0.94 (0.71-1.26) | 0.93 (0.69-1.26) | 0.43 | 0.77 |
| Values are ORs (95% CIs). | | | | | | | |
| Model 1 adjusted for age and energy intake. | | | | | | | |
| Model 2 adjusted for model 1 + education, smoking, physical activity, alcohol intake, sucrose, and fruits, berries, and vegetables consumption. | | | | | | | |
| * p-value for trend tested with linear regression represents the statistical significance of the linear trend across medians of consumption quintiles. | | | | | | | |
| ** Quadratic term p-value for trend across medians of consumption quintiles. | | | | | | | |
| Abbreviations: CI, confidence interval; g/d, grams per day; MetS, metabolic syndrome; OR, odds ratio; ref, reference group; SD, standard deviation. | | | | | | | |
