# Supplementary figures and images for: Fermented Dairy Consumption and Metabolic Syndrome in Finnish Men and Women
Source: J Nutr. 2026 Mar 2;156(5):101464. doi: 10.1016/j.tjnut.2026.101464 (PMC13197939; doi:10.1016/j.tjnut.2026.101464)

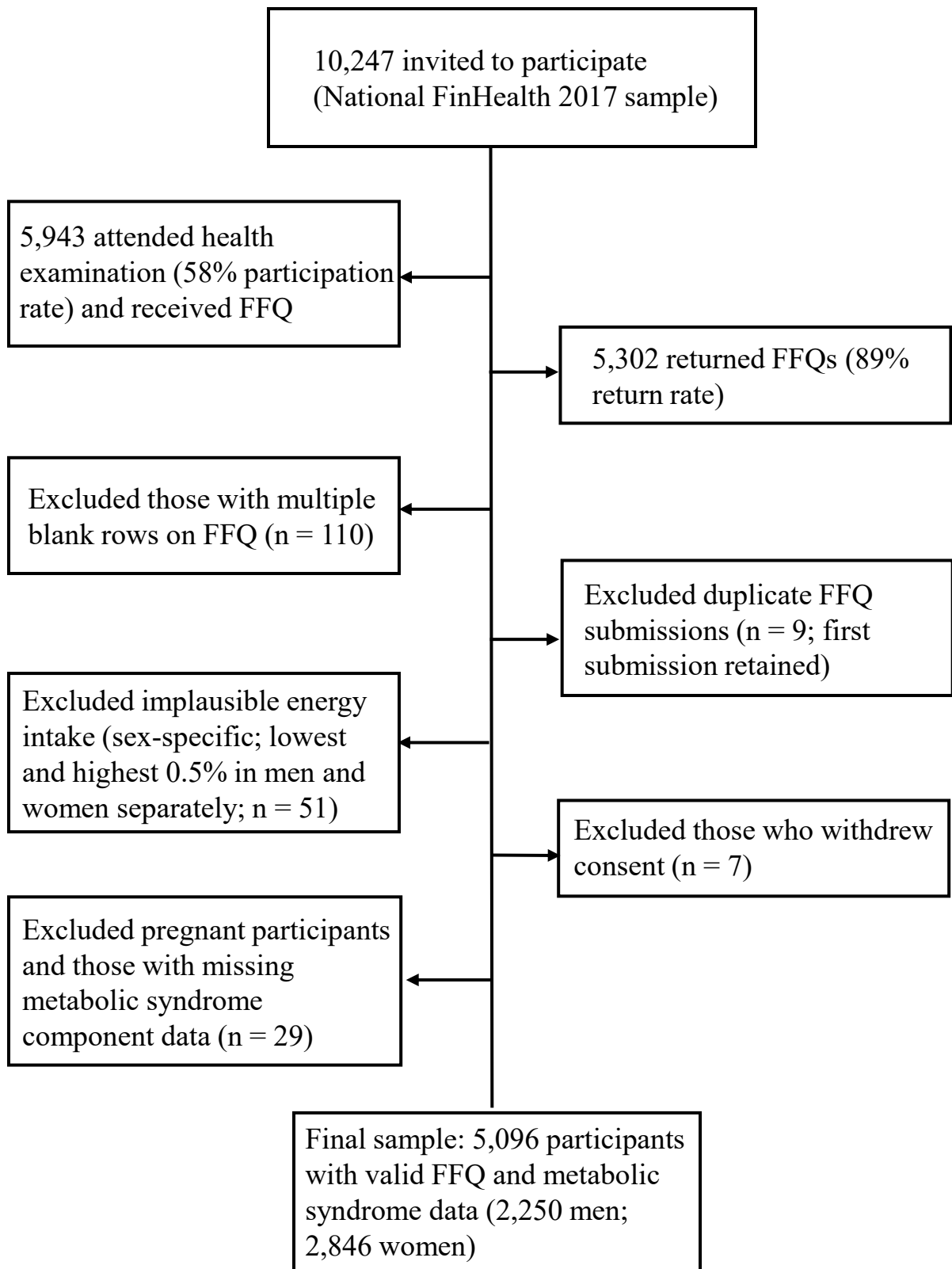

Supplement: Multimedia component 2 [file mmc2.pdf]
